# Experimental Study on the Physisorption Characteristics of O_2_ in Coal Powder are Effected by Coal Nanopore Structure

**DOI:** 10.1038/s41598-020-63988-4

**Published:** 2020-04-24

**Authors:** Bo Tan, Gang Cheng, Xiaoman Zhu, Xianbing Yang

**Affiliations:** School of Emergency Management and Safety Engineering, China University of Mining and Technology -Beijing, Beijing, 10083 China

**Keywords:** Surfaces, interfaces and thin films, Environmental, health and safety issues, Coal

## Abstract

Coal is a porous medium. Oxygen molecules in the air penetrate through the pores of coal and are adsorbed on the coal surface. Low-temperature oxidation of coal then occurs, by which coal spontaneous combustion is promoted. Given this process, the authors analysed the physisorption characteristics of O_2_ in pulverized coal from the perspective of nanopore structure. In this study, five different kinds of coal samples (two lignites, one bituminous coal, and two anthracites) were selected, and the surface morphology, pore structure parameters and oxygen physisorption capacity of the pulverized coals were determined by scanning electron microscopy (SEM), mercury intrusion porosimetry (MIP) and oxygen adsorption with chromatography (OAC), respectively. The experimental results of SEM and MIP show that with the development of coal, the surface folds increase, and the pores increase in number and shrink, which leads to the nanopores of anthracite and bituminous coal being smaller and more complex than those of lignite. The experimental results of OAC show that adsorbed oxygen is physisorbed by pulverized coal in the order lignite > bituminous coal > anthracite. Analysis of the oxygen desorption curves shows that the oxygen desorption rates of the anthracites and bituminous coal are slower than those of the lignites. The results show that the amount of oxygen physisorbed by pulverized coal is proportional to the fractal dimension of the coal pores, proportional to the pore volume of the nanoscale pores, and inversely proportional to the number of closed pores in the coal. Based on the results of the analyses mentioned above, it is important to analyse the process of coal-oxygen chemisorption and the mechanism for low-temperature oxidation of coal to prevent coal spontaneous combustion.

## Introduction

**Figure 1:**
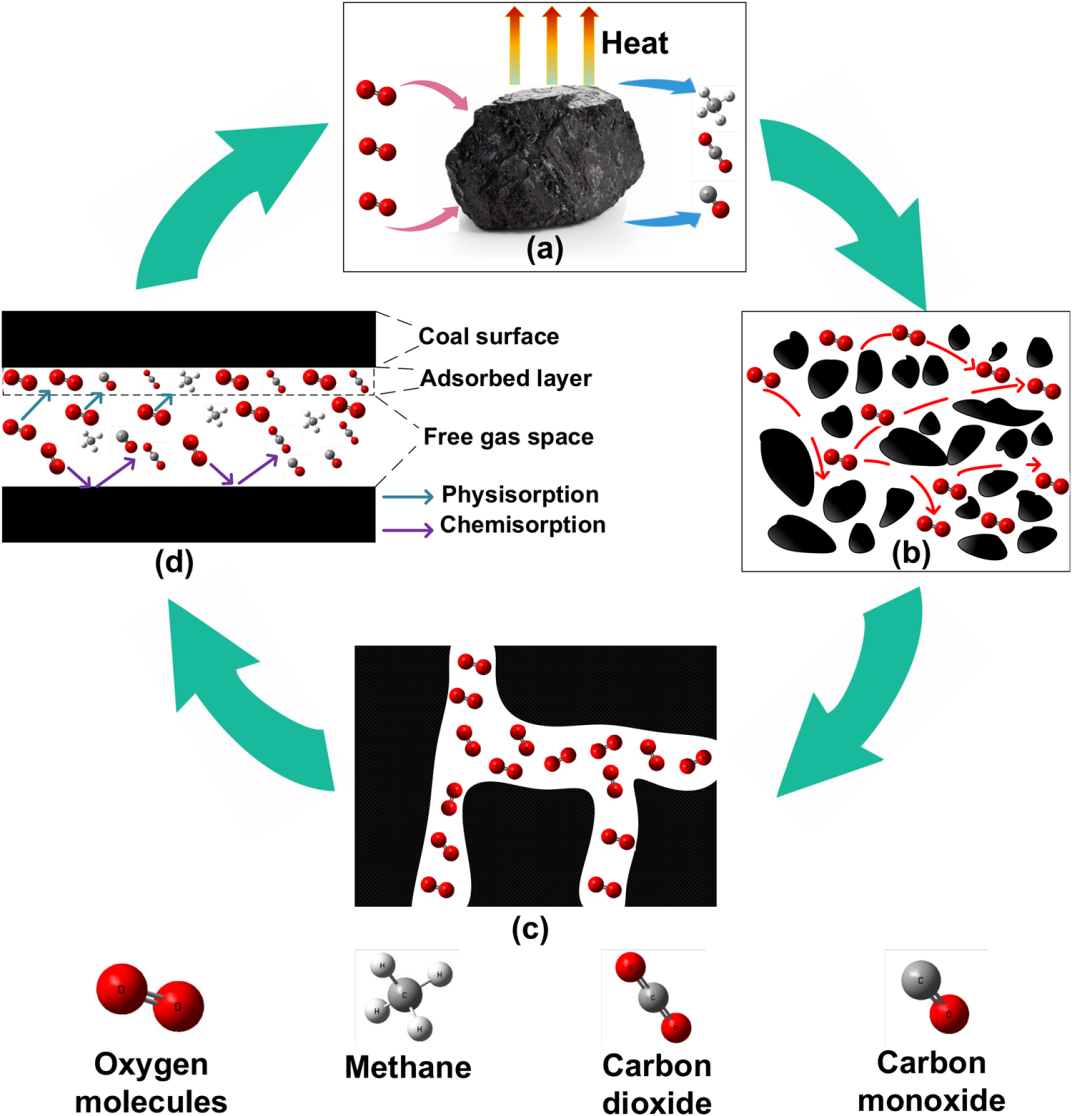
Diffusion and adsorption process of oxygen in the process of coal low-temperature oxidation.

The confirmed worldwide reserves of coal stood at approximately 1,055 billion tons in 2018. The United States, Russia, Australia and China together accounted for 66.1% of the world’s coal reserves^1^. During mining, storage and transportation, coal can undergo spontaneous combustion, resulting in a huge waste of resources and economic losses in the United States and China per annum. The initial stage of coal spontaneous combustion involves coal low-temperature oxidation, in which oxygen permeates into the coal through pores, then physisorbs and chemisorbs with the functional groups on the coal surface, and adsorption heat is generated and accumulated^2,3^, as shown in Fig. 1. Coal is a porous medium, and the pore structure of coal varies with coal development^4^; therefore, oxygen diffusion in coal pores and adsorption on coal surfaces are also different^5^. The significance of the coal low-temperature oxidation mechanism in the physisorption, chemisorption and diffusion of oxygen in coal nanopores has been studied. In this paper, all oxygen adsorption involved physisorption.

Scholars from various countries have performed much work on the influencing factors of coal low-temperature oxidation. At the macroscopic scale, the influences of moisture^6^, sulfur content^7^, particle size^8^, oxygen supply^9^ and other factors on the coal low-temperature oxidation process have been analysed. At the microscopic scale, Fourier Transform Infrared Spectrometer (FTIR)^10^, Nuclear Magnetic Resonance (NMR)^11^, Thermogravimetric Analysis (TG)^12^, quantum chemical calculation^13^, and applications in physical chemistry have been adopted to explain the mechanism of coal low-temperature oxidation. Tao *et al*. found that the heat generated by the decomposition of oxygen-containing functional groups in coal is the main reason for the initial temperature rise in coal spontaneous combustion oxidation^14^. Cai *et al*. analysed the variation in gaseous products and reaction characteristics of the main functional groups of different metamorphic coals during low-temperature oxidation, and the critical temperature for coal spontaneous combustion was determined^15^. Wang *et al*. analysed the mechanism of weight gain in the process of coal low-temperature oxidation through TG-FTIR^16^. Jingyu Zhao *et al*. analysed the critical points of the low-temperature oxidation stage of coal, including the critical temperature (97.45 ± 7.15 °C) and crack temperature (149.28 ± 8.32 °C)^17^; they analysed the exothermic characteristics of the functional groups of the coal during the oxygen adsorption and mass-increasing stages and believed that these two stages required the most caution and carried the most risks^18^. Buzhuang Zhou *et al*. analysed the free radical concentration, the volatile matter and the CO concentration of coal under different oxygen concentrations during the low-temperature oxidation stage and investigated the low-temperature oxidation mechanism of coal^19^. Ying Wang *et al*. analysed the functional groups that are responsible for low-temperature oxidation of coal at different temperatures^20^.

CO_2_, CO, CH_4_, N_2_, O_2_ and other gases permeate in coal pores, and the gas adsorption capacity of coal is affected by the adsorption and diffusion mechanism of gas in coal pores^21^. Scholars have analysed the gas adsorption capacity of coal in terms of pore type, pore shape, pore surface roughness, pore size distribution, specific surface area, total pore volume, and physical properties^22,23^. Scholars have performed much research on the adsorption and gas storage mechanism of coal-bed methane (CBM)^24–27^, while there is very little research on oxygen; most of these studies considered macroscopic aspects to explore the impact of oxygen adsorption factors^28–30^. Song *et al*. analysed the influence of intra-particle, inter-particle and external O_2_ diffusion on high-temperature heterogeneous oxidation^31^. Sampath *et al*. analysed the coal seam gas storage mechanism in the adsorption and hydrated states, and CH_4_-CO_2_ gas exchange in adsorption/diffusion has been proven to be a dynamic thermodynamic process^32^. Wu *et al*. used the Hwang model to build a shale gas adsorption and diffusion model in nanopores and combined it with the nanoporous bulk gas transport model to establish a shale gas nanoporous gas transmission model^33^. Song *et al*. analysed the effect of oxygen-containing functional groups and coal vitrinite on CO_2_/CH_4_/N_2_ double-competitive adsorption; the adsorption energies of pure CO_2_, CH_4_ and N_2_ are −31.69~−67.61 kcal/mol, −29.43~−46.83 kcal/mol and −14.57~−29.74 kcal/mol, respectively, indicating that CO_2_ has stronger adsorption than CH_4_ and N_2_ ^34^.

As shown above, scholars have performed some research on the oxygen adsorption of coal by considering the characteristics of coal nanopores. The characteristics of the coal nanopore structure have a significant effect on the adsorption of oxygen, thus affecting the coal low-temperature oxidation process. In this paper, the nanopore structural characteristics, the surface morphology and the adsorbed oxygen content of five coal samples (two lignites, one bituminous coal, and two anthracites) were determined experimentally, and the relationship between the coal pore structure characteristics and the coal physisorption and desorption of oxygen was comprehensively analysed. This study is of significance for analysing the coal low-temperature oxidation process and preventing coal spontaneous combustion.

## Materials and Methods

### Coal sample preparation

In this paper, five different kinds of coal samples are selected for analysis, and the samples are abbreviated as AN, HA, PI, SJ and IL. The raw coals were broken, ground and screened to ensure that oxygen could spread and adsorb in the coal pores, and coal powder samples with a particle size range of 0.154 mm to 0.180 mm were used in the experiments. Table 1 shows the basic parameters of the coal samples; the coal samples include anthracite, bituminous coal and lignite.

**Table 1:** Basic parameters of coal samples.

Coal sample	Industrial Analysis (%)				St,ad (%)	TRD (g/ml)	Coal type
	M_ad_	A_d_	V_daf_	FC_ad_			
AN	1.25	10.13	9.09	80.68	0.57	1.5544	anthracite
HA	1.01	12.64	4.86	82.28	1.48	1.5996	anthracite
PI	0.79	15.85	21.65	65.31	1.9	1.4532	bituminous coal
SJ	1.85	13.58	46.13	45.69	0.65	1.3563	lignite
IL	2.31	5.78	40.83	54.46	2.12	1.3428	lignite

Remarks: The moisture, ash and volatile carbon content were analysed by a Hunan Sude Company SDTGA5000a industrial analyser. The sulfur content was determined by a Hunan Sande SDS-V sulfur analyser.

### Experimental equipment and principles

The tests of the coal powder samples in this study were divided into two parts: the first part was designed to determine the surface morphology and nanopore structure of the coal powder, and the second part was designed to determine the oxygen adsorbed by the coal powder. The surface morphology was measured using a Hitachi S-4800 SEM, and the nanopore structure was measured by an Autopore 9510 automatic mercury intrusion meter produced by Micromeritics Corporation (United States). The adsorbed oxygen was measured by a ZRJ-1 coal self-ignition tendency meter produced by Beijing East-West Electronics Company.

#### Determination of coal powder nanopore structure

There are various methods to test coal nanopore structure^35–39^, which mainly include mercury intrusion porosimetry (MIP), low-pressure gas (N_2_/CO_2_) adsorption (LPGA), scanning electron microscopy (SEM), transmission electron microscopy (TEM), small-angle neutron scattering (SANS) and other technologies. The van der Waals diameter of the mercury atom is 0.32 nm, and the kinetic diameter of the oxygen molecule is 0.346 nm. The two values are similar; in MIP, mercury is pressed into pores in a high-pressure environment, similar to the diffusion of oxygen in pores with a certain air pressure. Therefore, it can be considered that the pore structure characteristics of pulverized coal measured by a mercury pressure instrument can be used to analyse the influence of the pore structure of the pulverized coal on gas diffusion and adsorption.

In this study, MIP was used to determine the coal powder nanopore structure, and the experimental instrument was an Autopore 9510 automatic mercury intrusion meter. The experimental instrument has two low-pressure ports and a high-pressure port. The low-pressure port has an analytical pressure range of 0.5 psi to 50 psi, the high-pressure port can analyse a maximum pressure of 60000 psi, and pore diameters from 3 nm to 4.4 × 10^5^ nm can be measured. A 3 cc powder dilatometer was used in the experiment, and mercury, sealing grease and seal oil were all provided by Micromeritics Corporation (United States).

The basic principle of MIP used to determine the size of coal powder pores is as follows: under immersion, mercury cannot enter pores without external pressure. The pores are assumed to be cylindrical in MIP, and the relationship between pressure and pore diameter can be obtained according to the Washburn formula^40^ as follows:

1 $$-p\pi {r}^{2}L=\gamma 2\pi rL\,\cos \,\theta =-\,p\Delta V$$

The relationship between pressure and pore radius can be obtained from the above formula as follows:

2 $$r=\frac{-2\gamma \,\cos \,\theta }{p}$$

In the above formula, the values of *γ* and *θ* are constant, and the relationship between pressure and the pore diameter is as follows:

3 $$r\,({\rm{nm}})=7570/p$$

4 $$D\,({\rm{nm}})=15140/p$$

where *r* is the pore radius; *D* is the pore diameter; *L* is the pore length, m; *γ* is the surface tension of mercury, 484 erg/cm; *θ* is the contact angle, 140°; *p* is the pressure, kgf/cm^2^; *S* is the pore surface area, m^2^; and Δ*V* is volume change in the injected mercury, m^3^.

#### Experiments of coal powder oxygen adsorption

**Figure 2:**
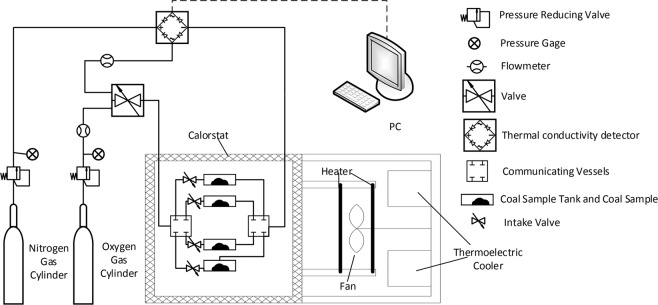
Simple diagram of the coal oxygen adsorption measuring device.

The experimental method used to analyse coal powder oxygen adsorption was OAC (according to the standard: GB/T 20104-2006), which is based on the adsorption of oxygen by coal under low temperature and normal pressure and assumes a theoretical basis of single-molecule physisorption. According to the Langmuir single-molecular-layer adsorption equation, the amount of oxygen adsorbed by coal under limited conditions is then determined. A simple illustration of the experimental device is shown in Fig. 2.

The experimental steps of coal powder oxygen adsorption were as follows. The volume of the coal sample tank, the instrument constant and the instrument correction factor were calibrated before the experiment. Coal powder was loaded into the coal sample tank, the valve was in the desorption state, the temperature of the thermostat was set to 105 °C, and the coal powder was desiccated for at least 1.5 hours (coal powder with less than 1% moisture was desiccated for 1.5 hours, and coal powder with more than 1% moisture was desiccated for 2 hours). Subsequently, the temperature of the thermostat was set to 30 °C, the valve was set to the adsorption state, and oxygen was adsorbed by the coal powder; after 20 minutes, the valve was adjusted to the desorption state, the thermal conductivity detector detected the desorbed oxygen, the varying voltage values were transmitted to the computer system for analysis, and the desorbed area of the loaded tank (*S*_1_) was calculated. Finally, the coal sample tank was emptied and filled with oxygen at 30 °C for 5 minutes, and the desorbed area of the empty tank (*S*_2_) was calculated after desorption. During the experiment, the flow rate of oxygen was controlled at 20 ± 0.5 cm^3^/min, and the flow rate of nitrogen was controlled at 30 ± 0.5 cm^3^/min. *S*_1_, *S*_2_ and other test parameters obtained from the experiment were substituted into formula 5, and the oxygen adsorbed by the coal powder could be calculated.

5 $${V}_{{\rm{d}}}=k\cdot {k}_{1}\cdot {R}_{c1}\left\{{S}_{1}-\left[\frac{{a}_{1}{R}_{c1}}{{a}_{2}{R}_{c2}}\times {S}_{2}\left(1-\frac{G}{{d}_{TRD}\cdot {V}_{s}}\right)\right]\right\}\times \frac{1}{(1-{W}_{d})\cdot G}$$

where *V*_*d*_ is the oxygen adsorbed by the coal powder, ml/g; *k* is the instrument constant, min/(mV·s); *k*_1_ is the instrument correction factor; *R*_*c*1_ is the loaded tank nitrogen flow, generally 30 ml/min; *R*_*c*2_ is the empty tank nitrogen flow, generally 30 ml/min; *a*_1_ is the ratio of oxygen partial pressure to atmospheric pressure in the loaded tank; a_2_ is the ratio of oxygen partial pressure to atmospheric pressure in the empty tank; *S*_1_ is the desorbed area of the loaded tank, mV·s; *S*_2_ is the desorbed area of the empty tank, mV·s; *G* is the weight of the coal sample, g; *d*_*TRD*_ is the true density of coal, g/ml; *V*_*s*_ is the sample tube volume, ml; and *W*_*d*_ is the moisture content of the coal sample, %.

## Results

### Surface morphology of coal

**Figure 3:**
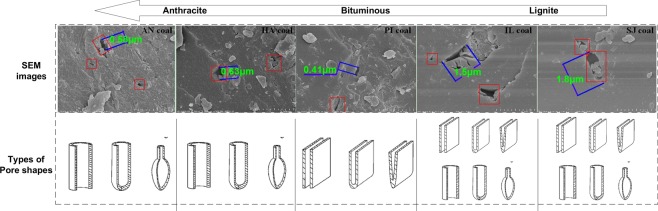
SEM images of 5 kinds of coal powder and their types of pore shapes.

As shown in Fig. 3, there are significant differences in the surfaces of different coal powders. The number of surface pores in the anthracite coal samples (AN and HA) and bituminous coal sample (PI) is more than two, but the pore diameter is less than 1 μm, and many nanoscale powders are attached to the surface. The number of surface pores (approximately 1 to 2) of the lignite coal samples (IL and SJ) is low, but the pore diameter is larger than 1 μm. From the surface analysis of the coal powders, the surface of the lignite is smooth and has few folds, the lignite coal has the most folds, and the bituminous coal has an intermediate number of folds.

Throughout coal development, the surface of coal powder is constantly compressed, large pores become small pores, the surface continuously forms folds, and the increase in folds increases the surface adsorption capacity of the coal^41^.

The types of coal pores include open pores and semi-closed pores^22^. Open pores are divided into cylindrical pores and parallel pores^42^. Semi-closed pores are divided into cylindrical pores, parallel-plane pores, wedge-shaped pores and ink-bottle pores^43^. As shown in Fig. 3, the pore shapes of each coal powder surface were assessed according to the SEM images: the pore sizes of IL and SJ are the largest, and there are more pore types; the pore types of PI are mostly parallel pores or wedge-shaped pores; and the pores of AN and HA are compressed into cylindrical pores or ink-bottle pores.

### Coal powder nanopore structure

The Dubinin pore classification standard is often used to study pore adsorption characteristics^42^. On this basis, Cai Yidong^35^, a Chinese scholar, proposed a pore classification method suitable for describing both adsorption and transport characteristics. Pores are divided into micropores (<2 nm), minipores (2~10 nm), mesopores (10~10^2^ nm), macropores (10^2^~10^3^ nm), super-macropores (10^3^~10^4^ nm) and microfissures (>10^4^ nm). According to the different abilities of gas adsorption and transportation with respect to pore diameter, pores can be divided into two types: adsorption pores (<100 nm) and seepage pores (>100 nm). The adsorption of gas by micropores is characterized as irreversible adsorption^35^. CO_2_, CO, CH_4_, N_2_ and other gases are adsorbed in the micropores, and desorption is difficult at very high temperatures^40^, so it is difficult for O_2_ to enter the micropores. Large pores also adsorb a small amount of gas due to the folds on the coal surface. Therefore, the main pore analysis in this paper focuses on minipores and mesopores.

Table 2 shows the experimental data related to the coal powder pore structure, as analysed by MIP. As coal continuously develops, the total pore volume, porosity and permeability of the coal decrease, and the average pore diameter, tortuosity and fractal dimension of the coal increase. Mainly because coal is constantly compressed in the development process, coal pores are constantly reduced in size, many minipores are compressed very little or not at all, and mesopores are compressed very little, resulting in more complex pores in anthracite than in other forms of coal^44^.

**Table 2:** Main experimental data from MIP.

Coal sample	Mercury intrusion data			Pore Structure			Coal type
	Total pore volume (ml/g)	Average pore diameter (nm)	Porosity (%)	Permeability (mdarcy)	Tortuosity	Fractal dimension	
AN	0.6493	380	46.6867	3465.7209	3.1749	3	anthracite
HA	0.6987	508	52.8272	3912.5922	3.0463	2.999	anthracite
PI	0.6667	302	49.6971	4064.7494	3.1762	2.997	bituminous coal
SJ	0.8149	123	53.3968	5236.6631	2.7536	2.996	lignite
IL	0.9466	83.9	56.0303	4990.9762	2.7401	2.995	lignite

**Figure 4:**
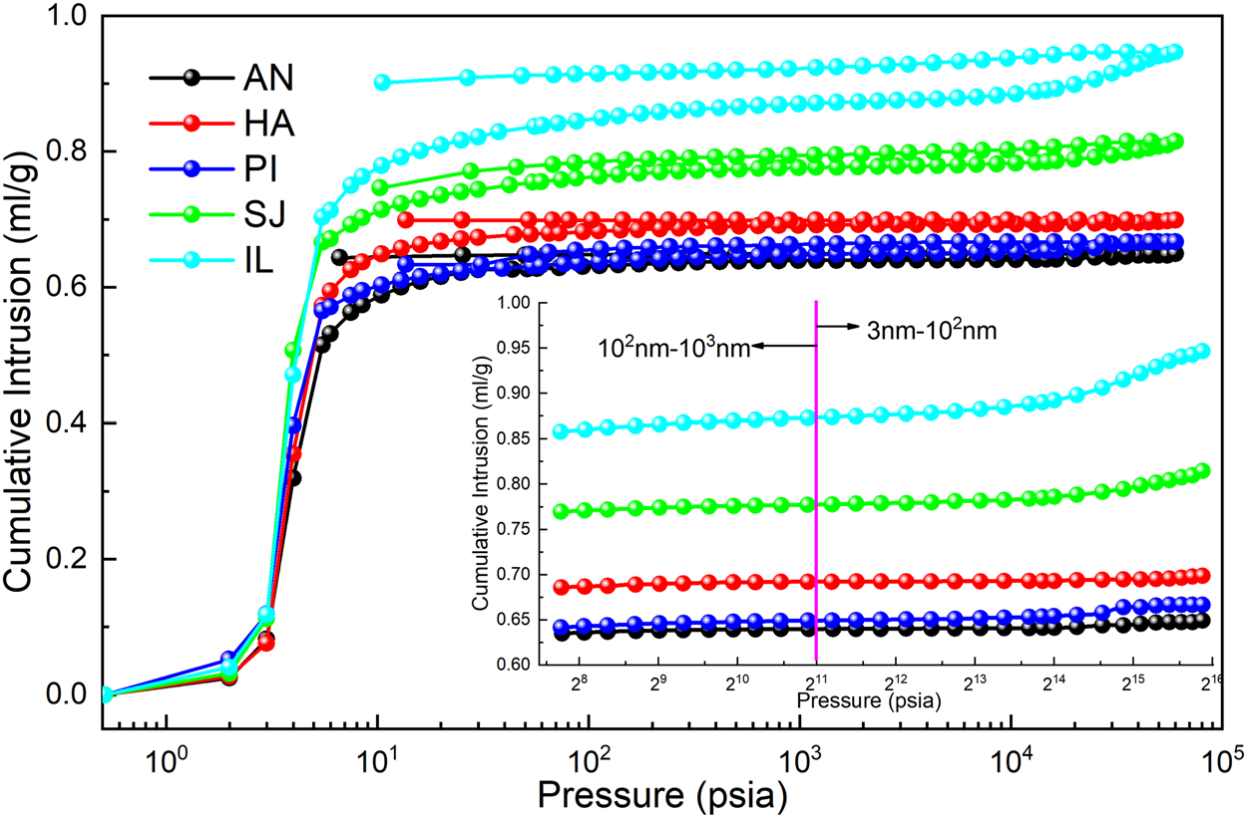
Coal sample mercury intrusion and mercury ejection curves.

**Figure 5:**
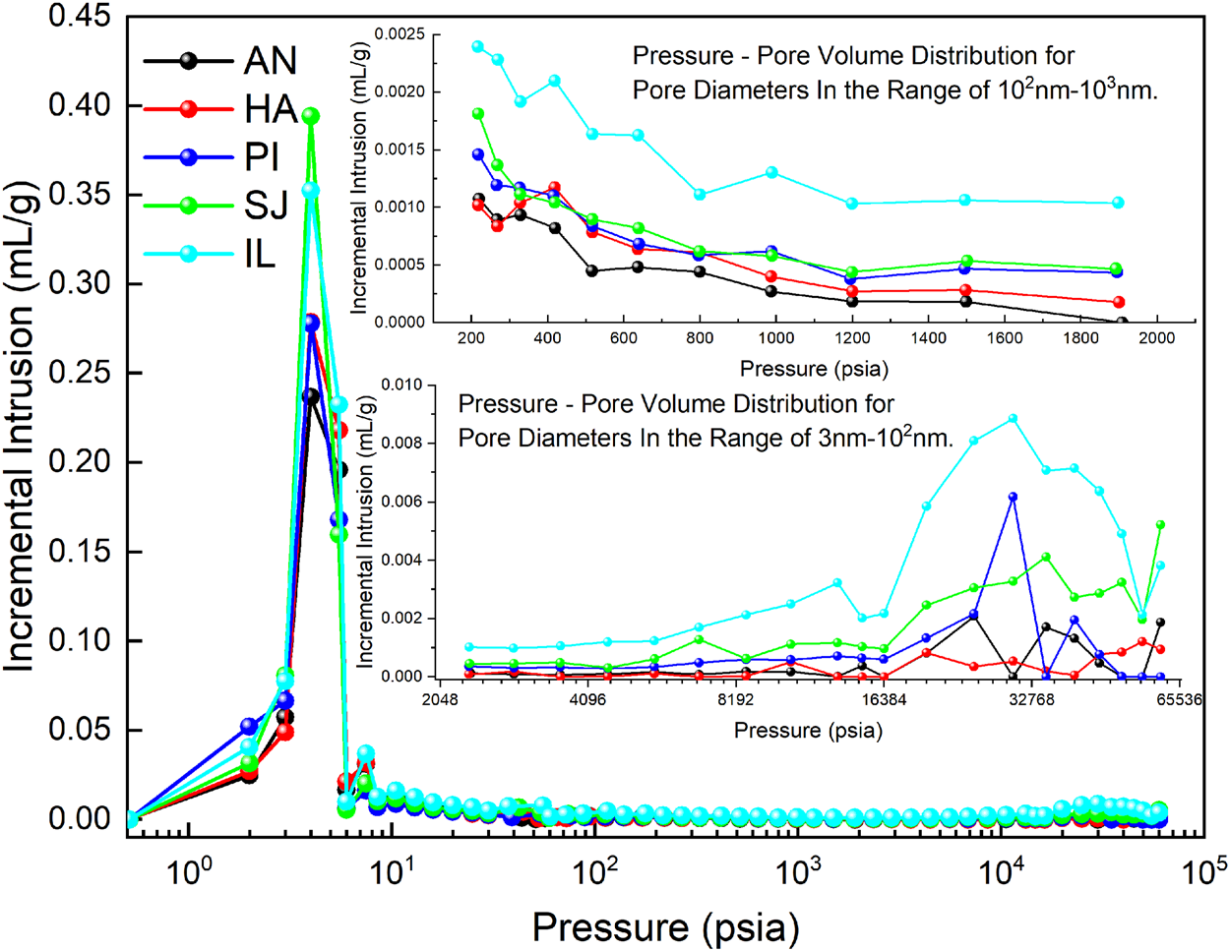
Coal sample pressure - pore volume distribution curves.

From the overall results in Fig. 4, mercury intrusion increases with pressure, and among the five coal samples, the pore volume of lignite is larger than that of anthracite and bituminous coal, as shown in Table 2. Pressures between 213 psia and 2134 psia (calculated according to formula (4)) represent 10^2^–10^3^ nm seepage pores, and the macropores in the two samples of lignite are larger than those in the samples of anthracite and bituminous coal, which means that gas flows and diffuses more easily in lignite. As shown in the mercury intrusion curve in the small figure in the lower right corner of Fig. 5, the lignite coal samples exhibit a greater increase in the amount of mercury intrusion in pores with a size below 100 nm than do the anthracite and bituminous coal curves. The amount of mercury intrusion corresponds to the pore volume, as shown in Table 3.

**Table 3:** Pore structure distribution of coal samples.

pore diameter/nm		macropores (100~1000)	mesopores (10~100)	minipores (3~10)	nanoscale pores (<100)	Total
AN	Pore Volume (ml/g)	0.0057	0.0022	0.0074	0.0096	0.0153
	Proportion (%)	37.25%	14.38%	48.37%	62.75%	100%
HA	Pore Volume (ml/g)	0.0072	0.0017	0.0049	0.0066	0.0138
	Proportion (%)	52.17%	12.32%	35.51%	47.83%	100%
PI	Pore Volume (ml/g)	0.0089	0.0065	0.0111	0.0176	0.0265
	Proportion (%)	33.58%	24.53%	41.89%	66.42%	100%
SJ	Pore Volume (ml/g)	0.0097	0.0109	0.0265	0.0374	0.0471
	Proportion (%)	20.59%	23.14%	56.27%	79.41%	100%
IL	Pore Volume (ml/g)	0.0175	0.0251	0.0484	0.0735	0.091
	Proportion (%)	19.23%	27.58%	53.19%	80.77%	100%

In the mercury intrusion curves, five coal samples have a hysteresis loop, and the hysteresis loops of the lignite coal samples are obvious, which indicates that there are many closed pores in lignite; gas that is adsorbed in closed pores is not easily excreted^45^.

As shown in Table 3, the pore volume of coal powder decreases with coal development. Analysis of the pore volume of coal pores with a size below 10^3^ nm revealed that the pore volume of the IL coal sample is nearly twice as large as that of the SJ coal sample and three to six times larger than those of the other three coal samples. In particular, the pore volume of the IL coal sample for pore diameters in the 3~10 nm range is 2 or even 10 times that of other coal samples. The pore volume of lignite minipores is relatively large, while the pore volumes of anthracite and bituminous coal minipores are small in comparison. Overall, the pore volume of lignite nanopores is larger than those of the other two coal samples.

### Oxygen adsorbed by coal

The adsorbed oxygen contents of the five coal samples were measured in four coal sample tanks each, as shown in Table 4. The adsorbed oxygen content of anthracite is more than those of lignite and bituminous coal. The average adsorbed oxygen contents of the AN coal sample and HA coal sample are 1.045 ml/g and 0.8625 ml/g, respectively. The average adsorbed oxygen contents of the PI coal sample, SJ coal sample and IL coal sample are 0.8575 ml/g, 0.7725 ml/g and 0.7975 ml/g, respectively. According to previous studies, the adsorbed oxygen content of coal follows the order anthracite > bituminous coal> lignite.

**Table 4:** Coal adsorption of oxygen.

Coal sample	Coal adsorption of oxygen (ml/g)				adsorbed oxygen (ml/g)
	No. 1 tank	No. 2 tank	No. 3 tank	No. 4 tank	
AN	1.04	1.06	1.04	1.04	1.04
HA	0.86	0.85	0.86	0.88	0.86
PI	0.82	0.96	0.82	0.83	0.82
SJ	0.72	0.75	0.8	0.82	0.72
IL	0.87	0.75	0.84	0.79	0.75

As shown in Fig. 4, there are many ink-bottle-type pores in lignite, and gas molecules cannot enter or desorb from ink-bottle-type pores. During the crushing process of coal blocks, the shape of the pores is destroyed, which causes the number of closed pores existing in different pulverized coals to be different. Therefore, different lignite powders may have different amounts of adsorbed oxygen.

**Figure 6:**
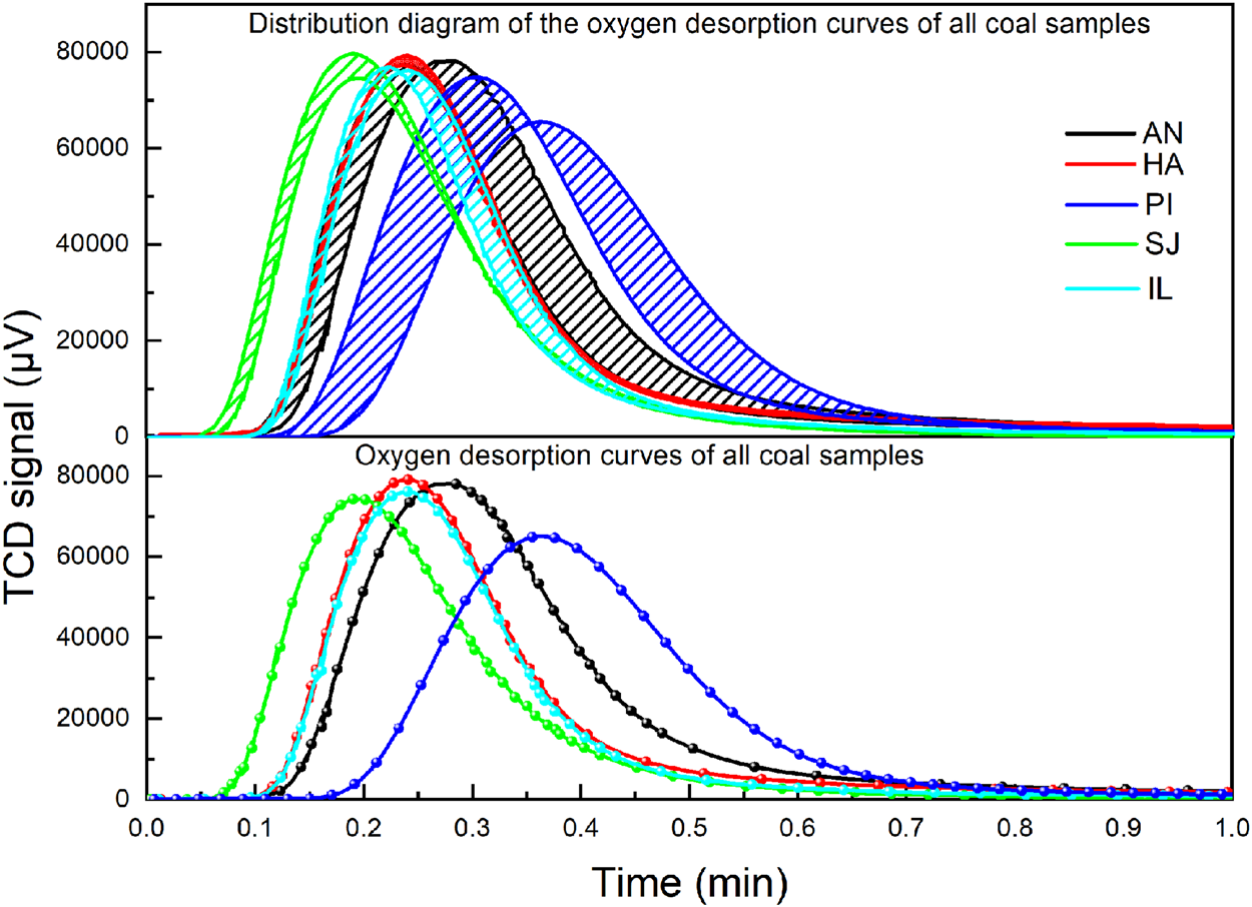
TCD detection results: oxygen desorption curves for coal samples after oxygen adsorption.

As shown in Fig. 6, the numerical fluctuations in the experimental results for AN, HA and PI are relatively small (the fluctuation in the No. 2 tank data for the PI coal sample was not considered). However, the numerical fluctuations in the experimental results for the SJ coal sample and IL coal sample are relatively large; these fluctuations have no correlation with the coal sample tank but have a certain relationship with the different coal powders in the coal sample tanks. The main reason for these results is that coal powder has closed pores; when pulverized coal is crushed, many closed pores in the coal are broken into open pores. Closed pores can affect the amount of oxygen adsorbed by coal; therefore, when different coal powders from the same coal sample were charged into the four coal sample tanks, the amount of adsorbed oxygen also differed, so the gap in the amounts of oxygen adsorbed by the lignite powder samples is large.

Since the oxygen adsorption data for the AN, HA and PI coal samples in the four sample tanks are relatively stable and close to the average values for the samples, the measured oxygen adsorption values are taken to represent the respective average values. For the SJ and IL coal samples, since the coal samples were first filled with nitrogen and then oxygen during the oxygen adsorption experiment, the pore structure of the coal samples was destroyed, which resulted in increased oxygen adsorption values for the SJ and IL coal samples. Therefore, their amounts of adsorbed oxygen were chosen to represent the minimum of the experimental results.

In summary, the amounts of oxygen adsorbed by the AN, HA and PI coal samples, 1.04 ml/g, 0.86 ml/g, and 0.82 ml/g, respectively, can be taken as the closest values to the average experimental value. As the SJ and IL coal samples experienced damage to the closed pores in the process of making pulverized coal, their adsorbed oxygen contents, 0.72 ml/g and 0.75 ml/g, respectively, are considered to be the minimum values of the experimental results.

As shown in Fig. 5, the oxygen desorption curve of the AN coal sample has a high peak, and the desorption time is longer than those of other samples. The peak of the oxygen desorption curve of the HA coal sample is the highest, and it lasts longer than those of the SJ and IL coal samples. The peak of the oxygen desorption curve of the PI coal sample is lowest, and the desorption rate is the slowest. The peak of the oxygen desorption curve of the SJ coal sample is low, and the desorption rate is first slow and then fast. The IL coal sample is similar to the SJ coal sample. The oxygen desorption curve of the lignite coal sample obtained from the TCD detection results is narrow, while those of the anthracite and bituminous coal samples are wide, indicating that the desorption of oxygen is faster in lignite than in anthracite and bituminous coal.

## Discussion

The characteristics of coal nanopores include the porosity, pore diameter distribution, pore volume, permeability, tortuosity, fractal dimension and other pore parameters, including the pore shape, which can be characterized by a cylinder, slit, wedge or ink-bottle morphology. These factors have a certain effect on the amount of oxygen adsorbed by coal.

### Influence of coal fractal dimension on oxygen adsorption

The coal pore structure parameters permeability, tortuosity and fractal dimension reflect the complexity of the coal structure. The complexity of coal structures can differ, which leads to different oxygen adsorption capacities of the coal surface. In particular, the fractal dimension is a comprehensive indicator of the degree of irregularity and complexity of the pore structure. The larger the fractal dimension is, the more complex the structure^46^.

**Figure 7:**
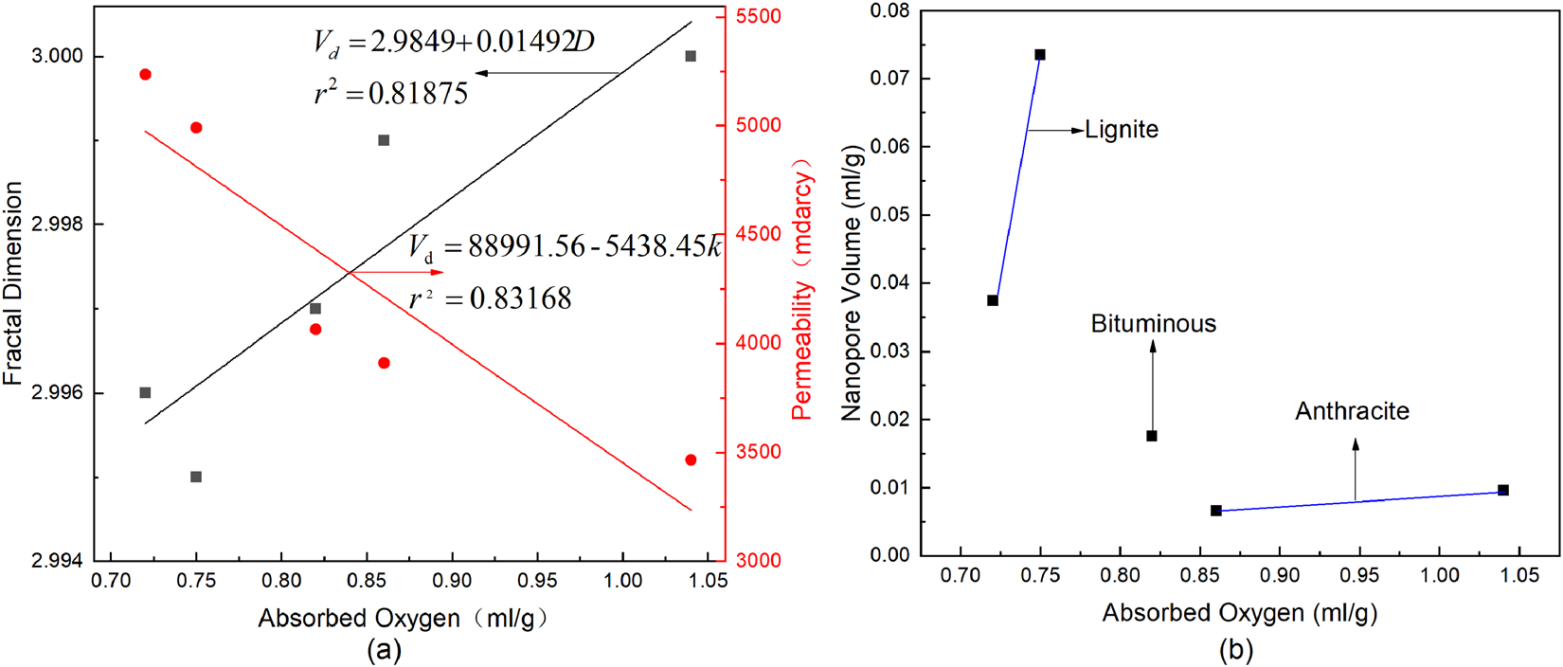
Relationship between the amount of oxygen adsorbed by coal and the pore structure of coal; (**a**) relationship between the amount of oxygen adsorbed by coal and both fractal dimension and permeability; (**b**) relationship between the amount of oxygen adsorbed by coal and nanopore volume.

As shown in Fig. 7(a), as the fractal dimension increases, the amount of oxygen adsorbed by the also increases. On the other hand, the fractal dimension of a coal sample also reflects the roughness of the coal surface. If the fractal dimension is larger, the surface of the coal body will be rougher, the specific surface area will increase, the number of oxygen adsorption sites provided by the pore surfaces of the coal body will increase, and the adsorption performance is will be enhanced. From the SEM images in Fig. 3, it can be seen that the surfaces of anthracite and bituminous coal are rougher than the lignite coal sample surface.

It can also be seen from Fig. 7(b) that as the permeability of coal increases, the amount of oxygen adsorbed by the coal decreases. As shown in Fig. 6, in the AN and PI coal samples, because of their low permeability and high fractal dimension, the coal adsorption force is strong, and the desorption process of oxygen is very slow. In contrast, the high permeability of the SJ coal sample corresponds to a low fractal dimension, which results in weak coal adsorption, and the desorption process of oxygen is faster.

### Influence of coal pore volume on coal adsorbed oxygen

Oxygen is mainly adsorbed in pores with a size below 100 nm. From the pore size data in Table 3 at the nanometre scale, the nano-scale pore volume of the AN coal sample is larger than that of the HA coal sample, indicating that the number of pores available for adsorption in AN is large, which causes the amount of oxygen adsorbed by the AN coal sample to be larger than that of the HA coal sample. Among the lignite coal samples, since the nano-scale pore volume of the IL coal sample is much larger than that of the SJ coal sample, the adsorbed oxygen content and the fractal dimension are inversely proportional. As shown in Fig. 7(b), the pore volume and the adsorbed oxygen content at the nanometre scale in the same type of coal are proportional to each other, and the relationship between pore volume and adsorbed oxygen in different types of coal is not obvious.

### Influence of shape characteristics of coal pores on oxygen adsorption by coal

**Figure 8:**
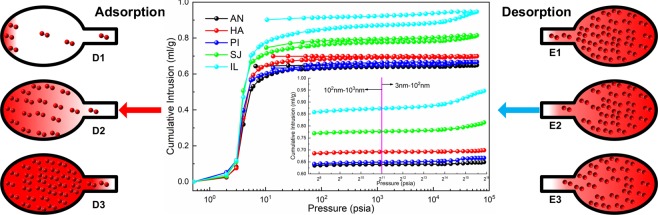
Gas adsorption and desorption in ink-bottle-type pores. The amount of mercury intrusion in IL coal samples at adsorption pressures of 3 psia, 10 psia, and 6e4 psia and at desorption pressures of 2.7 e4 psia, 3.3e3 psia and 10 psia.

As shown in Fig. 5, according to the shape of the “hysteresis loop” of the coal sample, the closed pores are ink-bottle type^40^; the SJ and IL coal samples have more ink-bottle-type pore structures, and most of the pores are nano-scale pores. An ink-bottle-type pore structure will cause a bottleneck effect, which will weaken the replacement of gas in the pores and the adsorption and desorption of gas by the pores^47^. A schematic of the adsorption and desorption process in ink-bottle-shaped pores is shown in Fig. 8, and data for the IL coal sample are provided as an example. Therefore, gas in the nano-scale pores of the lignite coal sample cannot be replaced by oxygen or cannot be desorbed from the pores during the desorption process, which is also the main reason for the low adsorbed oxygen content of lignite coal. Since the nano-scale pores in lignite cannot adsorb more oxygen, macropores (100–1000 nm) are the main places for oxygen adsorption in lignite coal samples. Because of the weak adsorption of macropores, only a single layer can adsorb oxygen, thus also leading to low oxygen adsorption by lignite.

### Additional influences

The adsorption of oxygen by coal is a result of multiple factors. In addition to the pore structure characteristics of the coal, the influences also include the coal physicochemical properties, microscopic composition characteristics and functional group composition and content. Previously published research was performed individually and is decentralized, and comprehensive analysis and evaluation are lacking. Therefore, an organic combination of various influencing factors was analysed here to determine their effect on coal oxygen adsorption.

## Conclusions

The pore structure characteristics of 5 coal samples (two lignites, one bituminous coal and two anthracite coals) were studied by MIP and SEM, and their adsorbed oxygen contents were determined by OAC. The effect of the coal nanopore structure on the oxygen adsorbed by the coals at room temperature (30 °C) was analysed. The main conclusions of this paper are as follows:

1. SEM provided the characteristics of the pore surface morphology of the coal powders. With the continuous development of coal, the surface folds of the coal become rougher, and the surface pores increase in number and shrink.
2. The pore structures of the 5 coal samples were determined by MIP. The experimental results show that anthracite and bituminous coal have fewer pores than lignite but their pore structures are more complicated. The lignite samples have large nanopore volumes (0.0374 ml/g and 0.735 ml/g), and the nanopore volume of the bituminous coal sample is medium (0.176 ml/g). The anthracite samples have small nanopore volumes (0.0096 ml/g and 0.0066 ml/g).
3. The lignite samples have more closed pores than the anthracite and bituminous coal samples, although their permeability is high and their pore connectivity is poor. The anthracite and bituminous coal samples have low permeability and a large fractal dimension, resulting in a rough pore surface, and oxygen is more easily adsorbed on the materials.
4. The amounts of oxygen adsorbed by the 5 coal samples were determined by OAC. The adsorbed oxygen contents of the anthracite samples are the highest (1.04 ml/g and 0.86 ml/g), while that of the bituminous coal sample is intermediate (0.82 ml/g), and those of the lignite samples are minimum (0.72 ml/g and 0.75 ml/g). The anthracite and bituminous coal desorption rates are slower than the lignite desorption rate.
5. The coal adsorbed oxygen content is proportional to the coal pore fractal dimension, proportional to the coal nanopore volume, and inversely proportional to the number of closed pores in the coal. The effect of the coal pore structure on the coal adsorbed oxygen content is the comprehensive result of multiple factors.
